# A Rapid Screening Method of Candidate Probiotics for Inflammatory Bowel Diseases and the Anti-inflammatory Effect of the Selected Strain *Bacillus smithii* XY1

**DOI:** 10.3389/fmicb.2021.760385

**Published:** 2021-12-17

**Authors:** Xuedi Huang, Fang Ai, Chen Ji, Pengcheng Tu, Yufang Gao, Yalan Wu, Fujie Yan, Ting Yu

**Affiliations:** National Engineering Laboratory of Intelligent Food Technology and Equipment, College of Biosystems Engineering and Food Science, Zhejiang University, Hangzhou, China

**Keywords:** inflammatory bowel disease, ulcerative colitis, probiotics, *Bacillus smithii*, lactic acid bacteria, zebrafish

## Abstract

Inflammatory bowel disease (IBD) is a chronic intestinal disease associated with the inflammatory gastrointestinal tract and microbiome dysbiosis. Probiotics are a promising intervention, and several probiotics have been reported to positively affect IBD remission and prevention, particularly on ulcerative colitis (UC). However, there is still a limitation in the knowledge of effectiveness and safety of probiotics therapies for IBD. Exploring more potential probiotics helps to find extensive evidence for probiotic intervention. This study established a rapid method for probiotics candidate screening and finally screened out one strain with the best protective effect. Forty strains isolated from four different sources were used for this screening. Hemolysis tests and acute toxic test evaluated strain safety. Zebrafish were first treated with dextran sodium sulfate (DSS) for colitis induction, and every bacteria were individually added to the fish water subsequently. Results showed eight strains could lower the larvae mortality within 3 days under a 0.6% DSS concentration, including *Lacticaseibacillus rhamnosus* GG, *L. rhamnosus* NBRC3425, *Bacillus smithii* DSM4216, *B. smithii* XY1, *Bacillus coagulans* NBRC12583, *Bacillus coagulans* XY2, *Lactobacillus parafarraginis* XYRR2, and *Bacillus licheniformis* XYT3. Among eight, *B. smithii* XY1 was the only strain having the equal ability to alleviate neutrophil infiltration in the larvae intestine with that ability of prednisolone under a 0.5% DSS concentration. *Bacillus smithii* XY1 restored intestinal epithelial cell integrity after DSS damage, as well as regulated the gene expression inflammation-related factors, indicating its bio-function of inflammatory response alleviation.

## Introduction

Inflammatory bowel diseases (IBD), types of chronic intestinal diseases characterized by immune-mediated intestinal inflammation of the gastrointestinal tract, affect millions of people every year (Ng et al., 2017). In recent decades, an increasing incidence rate has been characterized in newly industrialized countries, especially those transforming a western lifestyle, indicating that IBD has become a global disease in the 21st century (Ananthakrishnan et al., 2020). Due to the burden of the continuous rising IBD cases and growing health care costs, future therapeutics are suggested to achieve sustained durability of benefit and prevention and global sustainability, which is cost-effective, safe, and simple (Ananthakrishnan et al., 2020).

Probiotics are considered experimental interventions for functional symptoms of IBD (Colombel et al., 2019). Until now, the cause of the disease has still been hard to clarify. However, dysbiosis in the microbiota, mucosal immune system, and genetic factors play essential parts in the IBD’s pathogenesis (Fritsch and Abreu, 2019). Normal intestinal microbial flora is vital for the host to maintain the gut’s well-being and integrity of gut immunity (Zareef et al., 2020). In human subjects, the gut microbiome is different in patients with IBD compared with that in healthy control subjects (Glassner et al., 2020). The alteration of normal gut microbiota in the patients contributes to IBD incidence (Zareef et al., 2020). The host-microbial relationships lead to a sight of viable therapeutic approaches (Ni et al., 2017). Fecal microbiota transplantation (FMT) has been proved available not only in *Clostridium difficile* infection but also in some IBD clinical trials (Friedman et al., 2014; Browne and Kelly, 2017). Further evidence is needed before FMT or probiotics could be used in practice treatment, and probiotic or prebiotic therapies could be the next step (Friedman et al., 2014).

Contrary to most other therapeutics, multiple agent interventions in probiotics therapy show a low risk of harm (Colombel et al., 2019). Probiotics have succeeded widely in several IBD cases, especially in ulcerative colitis (UC), one of the two main entities of IBD (Basso et al., 2018). According to two double blind trials and mice model experiments, *Escherichia coli* Nissle 1917 has the equal ability of remission and prevention with mesalamine (Kruis et al., 1997; Rembacken et al., 1999; Kruis et al., 2004; Souza et al., 2020). Multiple lactic acid bacteria (LAB) and probiotic mixture also show positive effects, like *Lactobacillus acidophilus*, *Lacticaseibacillus rhamnosus* GG, *Bifidobacterium longum* 536, and VSL#3 (Zocco et al., 2006; Sood et al., 2009; Tursi et al., 2010; Wildt et al., 2011; Tamaki et al., 2016). Some strains have succeeded in some cases, while some strains are reported failed in clinical UC trials. The conclusion that extensive probiotics have beneficial effects on IBD patients is still controversial (Ng et al., 2010; Petersen et al., 2014). These failures suggest that the functional properties may be strain determined, encouraging establishment of new probiotics screening work and efficient methods for evaluating functions of different strains (Geier et al., 2007). Most research focuses on verifying the function of familiar strains like VSL#3 or *E. coli* Nissle (Kruis et al., 2004; Sood et al., 2009; Matthes et al., 2010). However, beneficial bacteria could come from various resources like food, air, soil, and animals (Zielińska and Kolożyn-Krajewska, 2018), though probiotics are initially believed to originate from the gastrointestinal tract of a healthy individual (Guarner et al., 2005). This study tried to isolate a wider range of anti-inflammatory bacteria from diverse resources, especially fermented foods. Fermented foods are promising bio-resources, especially those rich in multiple LAB. LAB are one of the most popular probiotics pools of probiotic therapies for diseases. For example, *Lactobacillus* and *Bifidobacterium* have been proved to be effective in clinical trials. So we used one commercialized probiotic, *L. rhamnosus* GG, to compare with the effect of other isolated strains on IBD improvement.

Multiple processing is required to finally define a microbe as a probiotic (Pereira et al., 2018). *In vitro* and *in vivo* safety test is the first step (Sanders et al., 2010). Using high-throughput experimental animal models with low cost is encouraged in screening lots of bacteria. Zebrafish has become one of the most common vertebrate models in cellular microbiology because of its benefits such as high throughput and ease of operation with simple immersion (Flores et al., 2020). The zebrafish gastrointestinal (GI) systems are highly homologous in functions and genes with the mammalian GI system. Over 100 IBD susceptibility genes found in IBD and zebrafish share homologous genes like NOD1, NOD2, e-cadherin, hnf4a, and ttc7a, suggesting it is a suitable animal model (Oehlers et al., 2011; Jostins et al., 2012; Zhao et al., 2018). Intestinal epithelial damage induced by chemical dextran sodium sulfate (DSS) mimics one of the critical features of IBD pathology. A DSS-induced IBD zebrafish model successfully represents the neutrophils infiltration at a medium 0.5% DSS concentration and high mortality at a high 0.6% DSS concentration (Oehlers et al., 2013, 2017). Acute mortality and the level of colitis inflammation set a scale for evaluating strains’ effectiveness.

In this study, we constructed a rapid isolating and screening method to efficiently isolate novel strains and expand the probiotics library for IBD remission or prevention. One novel strain with the best protective effect was discovered and underlying mechanisms were further investigated.

## Materials and Methods

### Bacterial Strains and Culture

#### Bacteria Strains and Culture

Forty *Bacillus* strains and LAB strains newly isolated and preserved in the laboratory were used. Twenty-one strains were isolated from fermented coffee grounds in Hangzhou, China. Seven strains were introduced by China Center of Industrial Culture Collection (CICC). Four strains were introduced by China General Microbiological Culture Collection Center (CGMCC). Three strains were isolated from dairy products and four from an “Effective Microorganisms” EM bacteria product (Supplementary Table S1). The isolation method is described in Supplementary Table S1. *Lactobacillus rhamnosus GG*, a strain widely used as a commercialized probiotic, was used as the reference strain.

#### Culture of Strains

Strains were grown for 18 h in suitable media and conditions in which they were isolated (Supplementary Table S1). All of the bacteria were subcultured three times before their use in the experiments. The bacterial broth was centrifuged at 12,000 rpm for 1 min at 4°C. Then, the precipitation was washed twice and resuspended with sterile PBS buffer. The suspension concentration was calculated with the Helber bacteria counting chamber (Auvon Helber Thoma; Rea et al., 2015, pp. 127–128).

#### Identification of the Strains by 16S-rRNA

Genomic DNA of the novel bacteria was extracted with TaKaRa No.9164, Japan, and 16S rRNA gene was amplified with Vazyme 2 x Phanta Max Master Mix, China. Sanger sequencing was conducted through the commercial service of Qingke, China. Gene sequences were analyzed in the BLAST Gene database. Phylogenetic analysis was constructed based on the 16S rRNA gene sequence through the neighbor-joining method by MEGA X software. The phylogenetic tree was plotted using EvolView.^1^ The 16S rRNA sequences have been deposited under GenBank NCBI (accession numbers and all strains used are shown in Supplementary Table S2). The number on a branch is the bootstrap value that indicates the extent of relatedness between two subjects.

### Zebrafish Manipulations

Adult zebrafish lines wild-type (WT: TU) and Tg (mpx:eGFP^il14^) were from core facilities, Zhejiang University school of medicine (CFZSM), and were kept there at a 14:10 light: dark cycle at 28°C. Fertilized embryos collected following natural spawning were cultured at 28.5°C in E3 water (5 mM NaCl, 0.17 mM KCl, 0.33 mM CaCl_2_, and 0.33 mM MgSO_4_) containing methylene blue (0.002 g/L). To observe fluorescent better at Tg (mpx:eGFP^il14^) fish line, an additional 75 μM 1-phenyl 2-thiourea was added into E3 water 24 h post-fertilization to prevent the pigment-cell formation. Methylene blue was removed 1 day before live imaging. Batches of 200 larvae were kept in sterile fish boxes in 80 ml E3 water, which was changed every day to 3 days post-fertilization (dpf). The Zhejiang University Laboratory Animal Center approved the protocols.

The protocol of DSS-induced intestine injury models was adapted and modified from Oehlers et al. (2013) and Chuang et al. (2019, p. 7). Fresh 0.6 and 0.5% (w/v) colitis grade DSS (36,000–50,000 MW) was prepared to induce high and medium levels of enterocolitis of 3 dpf larvae. All analyses were performed at 6 dpf unless otherwise noted.

### Safety Assessment of Strains

Hemolysis tests were used to do *in vitro* safety evaluation of the strains. Colonies were streaked on blood agar (10 g/L of tryptone, 10 g/L of NaCl, and 5 g/L of yeast extract; 100 ml/L of sterile defibrinated sheep blood was added after sterilization of the other medium components) plates with aseptic processing. Results were observed after 24 h of cultivation. γ hemolysis (lack of hemolysis; Savardi et al., 2018) means safe. For the *in vivo* safety test, the bacteria virulence assessment adapted from Ran et al. (2018, p. 3452) was done. Zebrafish larvae (4 dpf) were placed into 12-well plate (10 larvae per well) with 2 ml bacteria suspension (concentration of 1 × 10^6^ CFU/ml) of each bacteria strain for 3 days. *Escherichia coli* DH5α was used as a negative control. The larvae mortality was recorded after 3 days. Dead fish were removed from the wells during the experiments. Each experiment had three biological replications.

### Bacteria Screening

Each strain’s liquid culture was diluted into the same OD_600_ aseptically. Then, 100 μl of the culture dilution was inoculated into a 5 ml medium. After incubation, one strain with the highest final OD_600_ was selected from strains belonging to the same species isolated from the same sample. The strain culture was prepared at a concentration of 1 × 10^6^ CFU/ml. Prednisolone, an anti-inflammatory drug commonly prescribed for IBD treatment, was used as a positive control. Batches of 10 TU 3 dpf larvae were placed into each well (24-well plate) at a total volume of 2 ml 0.6% DSS E3 water with a final bacteria concentration of 1 × 10^6^ CFU/ml or 25 μg/ml positive drug prednisolone. A hit was defined as the higher mean value of survival compared to water control group. All first-round hits were rescreened with an inflammation assessment. Batches of 6 Tg (mpx:EGFP)^i114^ 3 dpf larvae were placed into 0.5% DSS E3 water. After 2 days of incubation, the 5 dpf larvae were washed with E3 water once and placed into bacteria or prednisolone dilution (same final concentration as above) for 24 h. The significant reduction of neutrophils infiltration was defined as a hit.

### Gene Expression Analysis

Total RNA used for real-time PCR from dissected intestines was isolated using RNAiso Plus (TaKaRa, Japan). Six larvae were used for one RNA extract. cDNA synthesis was performed using Prime Script™ RT reagent Kit with gDNA Eraser (TaKaRa, Japan). Real-time PCR was performed with the SYBR green method using TB Green Premix Ex Taq™ (TaKaRa, Japan). Triplicate PCRs were carried out for each sample analyzed. The data obtained were analyzed using StepOne Software v2.3. Modification of gene expression is reported concerning the control sample. The relative abundance of mRNA was determined by normalization to rpl13 levels, and the results were expressed as relative expression levels. The data were quantified by the comparative threshold cycle (ΔΔCt) method.

The primer sequences are rpl13 forward TCTGGAGGAC TGTAAGAGGTATGC, rpl13 reverse TCAGACGCACAATCTT GAGAGCAG, IL-1β forward GAGACAGACGGTGCTGTTTA, IL-1β reverse GTAAGACGGCACTGAATCCA, TNF-α forward CAGAGTTGTATCCACCTGTTA, TNF-α reverse TTCACGCT CCATAAGACCCA, IL-6 forward TCAACTTCTCCAGCGT GATG, IL-6 reverse TCTTTCCCTCTTTTCCTCCTG, NOD2 forward AGTTTCTGGGATTATGGGGT, an NOD2 reverse ACTGCCCACACCATTATCCA (Qin et al., 2018; Yi et al., 2019).

### Analysis of Neutrophilic Infiltration by Live Imaging

Six dpf larvae were anesthetized with 120 μg/ml tricaine for 30 s, mount larvae in 3% (w/v) methylcellulose for live imaging by epifluorescent microscopy (Oehlers et al., 2013). Inflammation level was indicated by the number of neutrophils congested in the intestine. Fluorescent neutrophils around the larval intestine were enumerated by manual counting. Larvae images were taken using a Nikon SMZ18 stereoscope.

### Hematoxylin and Eosin Staining

Zebrafish larvae were fixed in 4% paraformaldehyde overnight at 4°C and then dehydrated and embedded according to standard protocols. Transverse sections were prepared with LEICA RM2235 paraffin slicing machine and stained by auto Hematoxylin and Eosin (H&E) staining with Thermo GEMINI AS.

### Statistical Analysis

All analyses were performed using IBM SPSS Statistics 22. Differences were considered statistically significant when at *p* < 0.05. The mean ± SEM are used to display data. Neutrophil quantification data were analyzed using one-way ANOVA. RT-PCR data were analyzed using two-tailed Student’s *t*-tests for comparisons between the control group with other groups.

## Results

### Construction of a Strain Library

**Figure 1:**
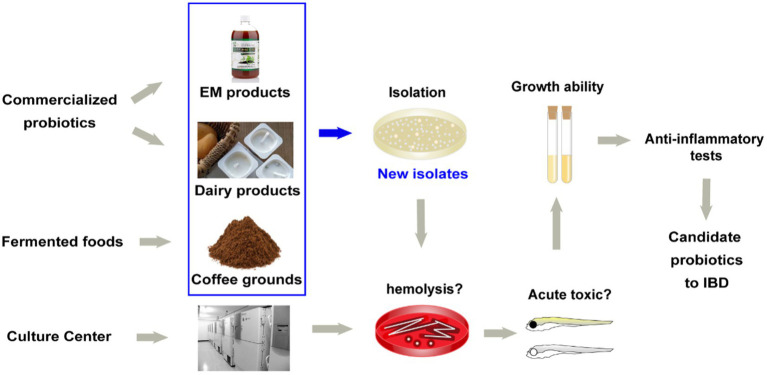
Source of strains and the screening method of candidate probiotics for inflammatory bowel diseases.

**Figure 2:**
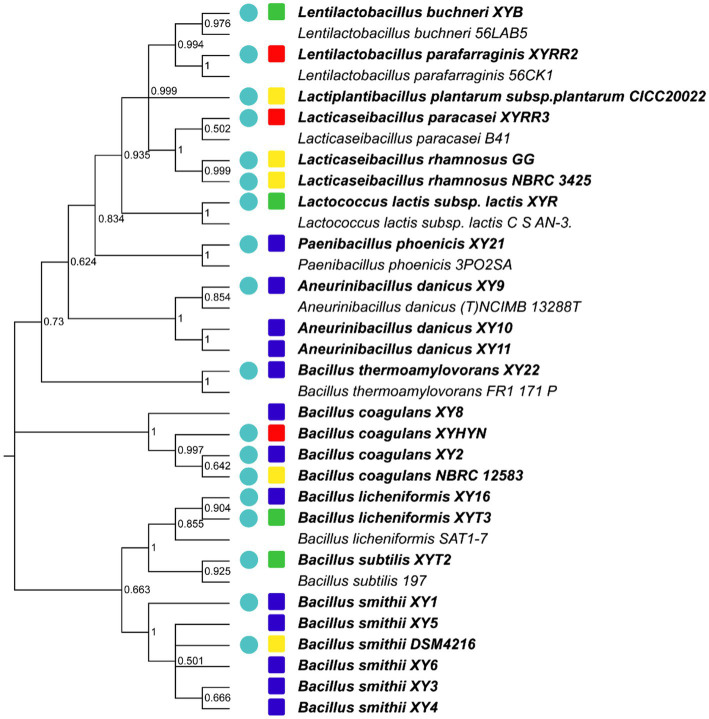
Phylogenetic relatedness of 16S rRNA between *in vivo* and *in vitro* safe strains isolated from different sources. Bold fonts mean strains used in this test. The circle indicates that the strain has the best growth ability from the same species and is isolated from the same sample. The colors of squares represent the sources of strains. Green, EM products; Red, dairy products; Yellow, culture centers; and Blue, coffee grounds.

The strains isolation strategy and the potential probiotics strain library were constructed as the implementation:

a. Bacteria isolation (Figure 1). More strains and more sources need to be considered. In this study, 29 novel strains were isolated from food samples (e.g., dairy products and fermented coffee grounds) and EM products (product contains multiple bacteria for agricultural usage; Table 1). Eleven other commercialized strains (from culture centers) were also included in the preliminary strain library.
b. Hemolysis test. Safety assessment of potential probiotics is vital. Hemolysis-positive strains are considered to have safety risks. In very rare immunosuppression, chronic diseases, or surgical interventions cases, several LAB strains of *L. rhamnosus* and some other *Lactobacillus* species, which caused human infection were detected to be hemolysis positive (Baumgartner et al., 1998; Figure 1). Among the preliminary strain library, 34 strains were hemolysis-negative (γ-lysis). The other six strains were hemolysis-positive (α-lysis or β-lysis; Table 1). All food samples and EM product isolates were hemolysis-negative, while nearly half of the strains from the culture centers were hemolysis-positive.
c. Acute toxic test. Being proved *in vivo* safety is another requirement of probiotics for medicine and food use. The high throughput zebrafish larvae model, a time-efficient and low-cost *in vivo* vertebrate model, was used to evaluate each strain’s virulence (Figure 1). Three-day exposure to 1 × 106 CFU/ml bacteria suspension of eight strains from coffee grounds resulted in larvae death. Other strains resulted in no mortality at the concentration of 1 × 10^6^ CFU/ml. The number of candidate strains in the lists was reduced to 26.
d. Growth ability test. All strains found *in vitro* and *in vivo* safety in this study were sequenced based on 16S rRNA. Most novel strains belonged to the genus *Lactobacillus* and *Bacillus*. Several new isolates from the same sample were also identified as the same species (Figure 2). These were defined as one group. One strain with the highest final concentration (represented by OD_600_) after incubation among the group was particularly picked out as a representative strain (Figures 1, 2). Strains with higher growth ability are prone to be easier to be commercialized. After the test, the probiotic strains in the library were reduced from 26 to 18. They are four from EM products, six from coffee grounds, five from culture center, and three from dairy products.

**Table 1:** Result of *in vivo* and *in vitro* safety assessment of strains.

Source	Strain ID	Hemolysis tests	Acute toxicity (survival %)
Coffee grounds	XY1	−	100 ± 0
	XY2	−	100 ± 0
	XY3	−	100 ± 0
	XY4	−	100 ± 0
	XY5	−	100 ± 0
	XY6	−	100 ± 0
	XY7	−	100 ± 0
	XY8	−	100 ± 0
	XY9	−	100 ± 0
	XY10	−	100 ± 0
	XY11	−	100 ± 0
	XY12	−	3 ± 1
	XY13	−	3 ± 1
	XY14	−	3 ± 1
	XY15	−	3 ± 1
	XY16	−	100 ± 0
	XY17	−	90 ± 0
	XY18	−	90 ± 0
	XY19	−	90 ± 2
	XY20	−	73 ± 3
	XY21	−	100 ± 0
	XY22	−	100 ± 0
Culture Collection	LGG	−	100 ± 0
	CICC 20022	−	100 ± 0
	NBRC 3425	−	100 ± 0
	CICC 10309	+	−
	CICC 23632	+	−
	CGMCC 1.4261	+	−
	CICC 10580	+	−
	CICC 23057	+	−
	CICC 10061	+	−
	NBRC 12583	−	100 ± 0
	DSM 4216	−	100 ± 0
EM product	XYB	−	100 ± 0
	XYR	−	100 ± 0
	XYT3	−	100 ± 0
	XYT2	−	100 ± 0
Fermented dairy product	XYRR3	−	100 ± 0
	XYRR2	−	100 ± 0
	XYHYN	−	100 ± 0

### Strains Screening for Mortality Reduction of Acute Colitis

**Figure 3:**
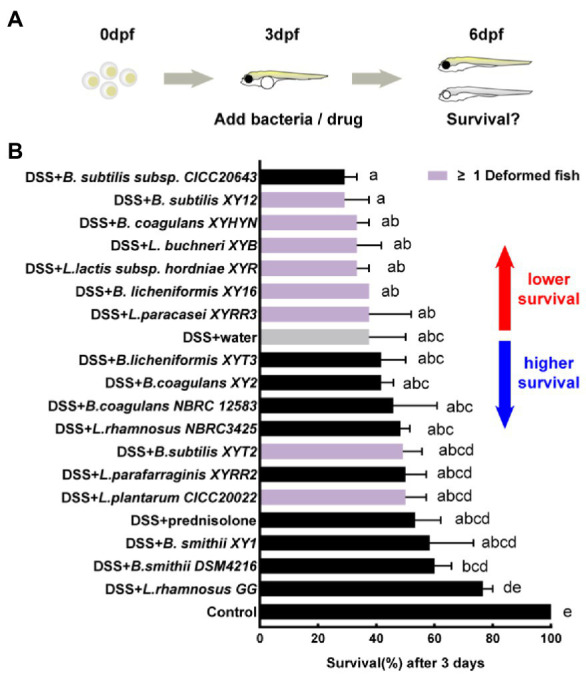
First hits on reducing mortality of acute colitis. **(A)** Schematic depicts the screening methodology to reduce the mortality of 0.6% dextran sodium sulfate (DSS) induced acute colitis. **(B)** Survival of larvae after 1-day immersion of bacteria suspension, data are presented as mean ± SEM, *n* ≥ 8 biologically independent animals from three independent experiments. Strain is considered an effective candidate when the mean survival rate exceeds water control (gray). Values indicated by the bars with different letters are significantly different (*p* < 0.05, one-way ANOVA).

The DSS-induced zebrafish acute colitis model has a dose-effect relationship for different mortality levels and intestinal inflammation (Oehlers et al., 2013). A high concentration of 0.6% DSS was first added to record the survival of larvae because acute mortality is more straightforward to observe than live imaging (Figure 3A). The 0.6% DSS dose results in 62.5% mortality in larvae after 3 days of exposure (Figure 3B). Prednisolone, the commercial drug for the treatment of IBD, reduces the mortality of larvae. The first anti-inflammatory test identified eight hits with higher survival and causing no malformation of larvae compared to the negative control, namely, *L. rhamnosus GG*, *B. smithii* DSM4216, *B. smithii* XY1, *L. parafarraginis* XYRR2, *L. rhamnosus* NBRC3425, *B.s coagulans* NBRC 12583, *B. coagulans* XY2, and *B. licheniforms* XYT3. Other strains may have side effects like edema (or different deformation results) or severe toxicity, causing greater mortality. Among them, two strains (*L.s plantarum* CICC20022 and *B. subtilis* XYT2) independently caused malformations. One strain (*B. subtilis subsp*. CICC20643) increased mortality. Furthermore, six strains (*L. paracasei* XYRR3, *B. licheniformis* XY16, *L. lactis subsp. hordniae* XYR, *L. buchneri* XYB, *B. coagulans* XYHYN, and *B. subtilis* XY12) caused both side effects (Figure 3B).

### Effects of Different Strains on Medium Intestinal Inflammation Alleviation

**Figure 4:**
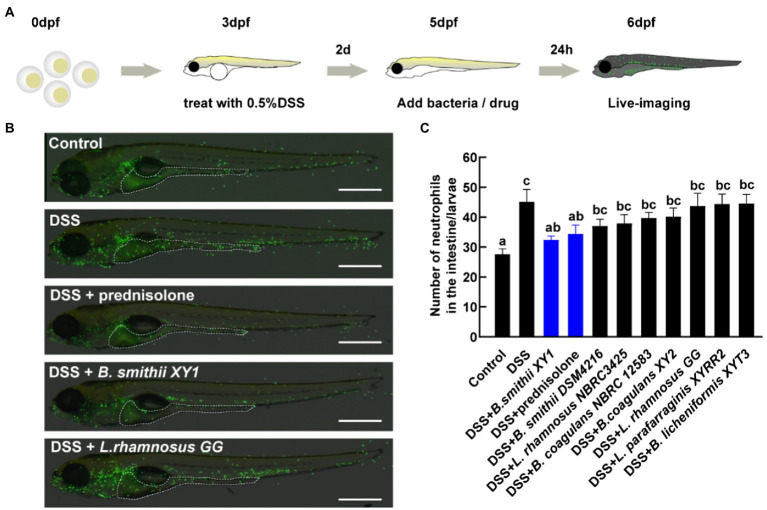
Second hits on reducing intestinal inflammation. **(A)** Schematic depicts the screening methodology to suppress neutrophilic inflammation. **(B)** Representative images of live imaging in 6 days post-fertilization (dpf) Tg (mpx:EGFP)^i114^ larvae exposed to 0.5% DSS at 3 dpf and treated with a drug and bacteria at 5 dpf. The area of the intestine is illustrated by a white dotted line, scale bars, 500 μm. **(C)** The number of neutrophils in the intestine per larvae, data are presented as mean ± SEM. Values indicated by the bars with different letters are significantly different (*p* < 0.05, one-way ANOVA). *n* ≥ 8 biologically independent animals from three independent experiments.

The eight hits from the first anti-inflammatory test were subsequently further screened. Larvae were kept under a condition at a lower concentration of 0.5% DSS, which caused lower mortality (less than 2%) and a medium level of intestinal inflammation (Figure 4A). The localization of neutrophils in the intestine was assessed by live imaging of Tg (mpx:EGFP)^i114^ larvae (Figure 4B). Among the strains that increased the survival rate of zebrafish damaged by a relatively high dose DSS, *Bacillus smithii* XY1 was the only one to significantly reduce neutrophil mobilization. Its improvement effect is equivalent to prednisolone (Figure 4C). Other strains have no significant impact on inflammation alleviation.

### *Bacillus smithii* XY1 Rescues Abnormal Intestinal Features in the IBD Zebrafish Model

**Figure 5:**
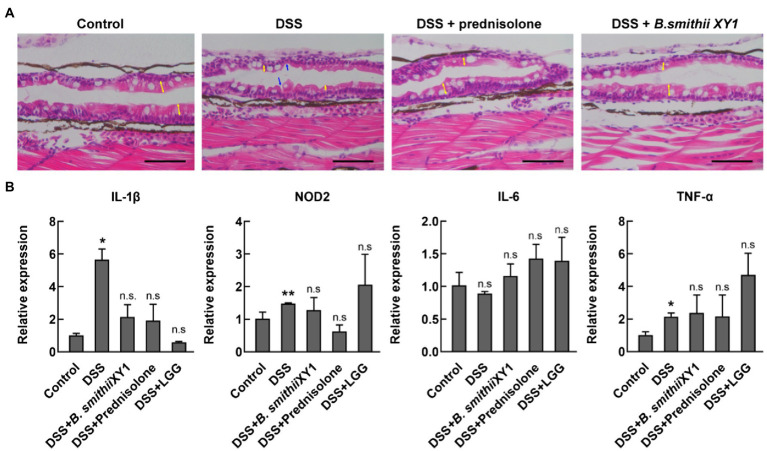
*Bacillus smithii* XY1 protects the intestine epithelium. **(A)** Hematoxylin and eosin (H&E) staining of a longitudinal section of zebrafish larvae intestine, scale bars, 50 μm, and yellow arrows indicate the length of the villi. Blue arrows indicate obvious gaps because of the thinner mucosal layer. **(B)** The mRNA level of immune-related genes including IL-1β, NOD2, IL-6, and TNF-α in the 6 dpf larvae. Data are presented as mean ± SEM. ^*^p < 0.05 and ^**^p < 0.01, analyzed by two-tail test. Every character represents the significance of a treatment group compared to the control group. *n* = 6 from three independent experiments.

Exposure to DSS for 2 days has been documented to cause gross changes to intestinal morphology. Zebrafish sections were taken longitudinally along the zebrafish mid and posterior segments of the 6 dpf larval intestines. Sections were assessed for villus structure, a clear and discernable epithelial monolayer, intestinal epithelial cell integrity, and the presence of mature goblet cells with large secretory vesicles (Jardine et al., 2019). Abnormal intestinal phenotypes, such as shorter and thinner villi, were observed in the DSS-induced model, resulting in apparent gaps. Other abnormal intestinal phenotypes include smaller goblet cells and signs of apoptosis (Figure 5A). Rescue of these changes with *B. smithii* XY1 and prednisolone was observed.

### *Bacillus smithii* XY1 Effects on Disturbed Innate Immune Genes Expression

Expression levels of the inflammatory cytokine gene IL-1β and TNF-α in the DSS group were significantly higher than in the negative control. The expression of the two genes was not significantly different in *B. smithiii* XY1, LGG, and prednisolone intervention compared with the control (Figure 5B). IL-6 gene expression was not significantly different in the treatment groups and the control group. NOD2 is a vital gene that is closely related to IBD pathogenesis. The NOD2 gene was highly expressed in the DSS group but similarly expressed in *B. smithiii* XY1, LGG, and prednisolone intervention groups. Though the TNF-α and NOD2 gene expression of the *B. smithiii* XY1 group was not significantly different from and LGG group, the mean of the LGG group is higher than the mean of the *B. smithii* XY1 group, which may relate to the result of the difference in Figure 4C.

## Discussion

A rapid and preliminary bacteria screening approach for IBD remission was designed for this work. The methodology involves six main steps: isolation, hemolysis test, acute toxic test, growth ability test, lethality reduction test for acute colitis, and reducing inflammation test. It takes care of both safety and function, and more safety assessments should be included in the screening process when considering uncommon bacteria species (Pereira et al., 2018). The approach is exceptionally suitable for labs possessing zebrafish infrastructure. Screening work, especially the acute toxic and anti-inflammatory tests, consumes many model animals. The high-throughput characteristic of the zebrafish larvae model meets the need. When we were conducting the methodology, coffee grounds were presented to be a promising bio-resource. Among the 22 novel strains isolated from coffee grounds, 14 were proven preliminary *in vitro* and *in vivo* safe, and 50% of best-grown strains passed the first screening to reduce acute colitis mortality. Although only *Bacillus smithii* XY1 isolated from coffee grounds passed the final screening, others are still worth further exploitation, especially those Generally Recognized as Safe (GRAS) or the qualified presumption of safety (QPS) recommended strains. *Baciillus smithii* XY1 also shows good tolerance to acid, especially bile (Supplementary Figure S1), suggesting that *Bacillus smithii* XY1 is prone to survive well in the intestine environment. It is better to include the acid and bile tolerance assessment after the growth ability test, but it is unnecessary. Live or dead bacteria may both have functions.

As a food-originated bacteria pool, dairy products present one strain of *L. parafarraginis* XYRR2, reducing mortality of acute colitis. EM products and their bacteria may not be suitable for anti-inflammation use because no strain from EM products presents effectiveness in the final two screening steps. This study also suggests fermented foods are treasuries for finding new IBD remission probiotics, though the number of tested sample strains was limited. Searching for candidates in a culture center is also a good option. Bacteria from the culture centers may be another probable candidate probiotic resource for IBD remission, particularly LAB species like *L. rhamnosus*, *B. smithii*, and *B. coagulans*, except some *L. licheniformis* strains. Nevertheless, an effort is needed to draft lists, which could be more costly in expense and time.

Protective effects of bacteria in the IBD zebrafish model are found to be strain-determined. Over 50% of bacteria used in the first anti-inflammatory test were beneficial. However, the other seven strains do not have anti-inflammatory effects and even result in harmful effects like lower survival and deformation. The inner differences within the strains also exist. For species like *B. coagulans* and *L. licheniformis*, *B. coagulans* XY2 and *L. licheniformis* XYT3 have positive effects on reducing acute colitis mortality in zebrafish models, but *B. coagulans* XYHYN and *L. licheniformis* XY16 could result in lower survival and deformation. There is a risk to using probiotics casually in the host with a damaged gut without testing first. This suggests that probiotics treatment should be more careful and we should regulate the specific strains used in IBD therapies.

Lactic acid bacteria were presented to be beneficial in anti-inflammation function again. Active strains like *L. rhamnosus* and *B. coagulans* were found to be anti-inflammatory in mice models already (Yan et al., 2017; Shinde et al., 2020). In this experiment, we confirmed their anti-inflammatory function in the DSS-induced acute colitis zebrafish model. On the other hand, *L. parafarraginis* and *B. smithii* are rarely reported as anti-inflammatory bacteria. This study provides evidence for these two species in exploring their bio-functions.

*Bacillus smithii* XY1 is found to be especially effective in anti-inflammation compared with any other strain. In this study, *B. smithii* XY1 is the only strain that could both reduce the mortality in acute colitis zebrafish models and significantly relieve inflammation in the intestine. *Bacillus smithii* XY1 used to be classified as *B. coagulans* until the late nineteenth century, suggesting that characteristics like oral safe and some immunomodulatory function of *B. coagulans* could also come from *B. smithii* (Nakamura et al., 1988). Species *B. smithii* has been added to the list of QPS-recommended biological agents intentionally added to food or feed as notified to EFSA since 2017 (Ricci et al., 2017). Another *B. smithii* strain has also been reported to present some anti-inflammatory function on animal models recent years. *Bacillus smtihii TBMI 12* is proved to protect against *C. difficile* infection in mice, and its spores protect against the Salmonella serotype enteritidis in mice, though its protective mechanisms have not been described (Jõogi et al., 2007; Suitso et al., 2007). Combining the former research on *B. smtihii TBMI 12* and our study, we found that *B. smithii* show its probiotics potential on anti-inflammation.

*Bacillus smithii* XY1 might be involved in immune regulation. RT-qPCR analysis shows gene expression of pro-inflammatory cytokines IL-1β and TNF-α could be restored to normal level after *B. smithii* XY1 intervention. The two cytokines are involved in NF-κB and IRF pathways. Unmoral high expression of these two genes reflects the inflammation in the host. However, whether *B. smithii* XY1 reduces inflammation levels by downregulating these genes expression directly is still unverified, and this needs further study. NOD2 also participates in the NF-κB signaling pathway. NOD2 is an innate immune sensor. It encodes a protein that helps NF-κB be responsive to bacterial lipopolysaccharides (Ogura et al., 2001; Strober and Watanabe, 2011). The changes of NOD2 gene expression levels may suggest that lipopolysaccharides change in the inner gut microbiome, which may lead a way to explore the mechanism of anti-inflammatory function of *B. smithii* XY1. Moreover, *B. smithii* XY1 helps the host to restore the intestinal epithelial cell integrity after DSS damage. Integral intestinal epithelial construction helps the host to insulate harmful microbes from the body.

As a promising and sustainable potential therapy of IBD, probiotics therapies still need efforts to find more effective strains and study the relationship between the probiotics, the host, and the gut microbiomes. Conventionally, human gastrointestinal is recommended as the source of probiotics by FAO and WHO. However, probiotics may also be isolated from unconventional sources. The physiological structure of zebrafish still differs from rodents and humans, so it is better to confirm strains’ beneficial effects in other models. Broader species screening would help set up a larger potential IBD remission probiotics library. Not only LAB but also some uncommonly used *Bacillus* like *B. smithii* and *B. licheniformis* presented to be anti-inflammatory. For specific strains, the mechanisms that their better anti-inflammatory effects differ from other strains of the same species are worth studying.

In conclusion, we firstly established a rapid method for choosing suitable bacteria and screened out one novel strain *Bacillus smithii* XY1 that successfully worked against DSS-induced intestinal damage in zebrafish larvae. *Bacillus smithii* XY1 intervention alleviated inflammatory response including neutrophil mobilization reduction, abnormal intestinal phenotypes rescue, and downregulation of inflammatory cytokine genes’ expressions. The present work has demonstrated the excellently protective effects of *Bacillus smithii* XY1 on intestinal inflammation recovery in the zebrafish model, which is meaningful for application in IBD therapy.

## Data Availability Statement

The datasets presented in this study can be found in online repositories. The names of the repository/repositories and accession number(s) can be found in the article/**Supplementary Material**.

## Ethics Statement

The animal study was reviewed and approved by Zhejiang University Laboratory Animal Center.

## Author Contributions

XH: conceptualization, methodology, writing – original draft preparation, and writing – reviewing and editing. FA and CJ: investigation. PT, YG, and YW: supervision. FY: supervision and writing – reviewing and editing. TY: project administration and funding acquisition. All authors contributed to the article and approved the submitted version.

## Conflict of Interest

The authors declare that the research was conducted in the absence of any commercial or financial relationships that could be construed as a potential conflict of interest.

## Publisher’s Note

All claims expressed in this article are solely those of the authors and do not necessarily represent those of their affiliated organizations, or those of the publisher, the editors and the reviewers. Any product that may be evaluated in this article, or claim that may be made by its manufacturer, is not guaranteed or endorsed by the publisher.

## Footnotes

^1^ https://www.evolgenius.info/evolview/

## Supplementary Material

The Supplementary Material for this article can be found online at: https://www.frontiersin.org/articles/10.3389/fmicb.2021. 760385/full#supplementary-material
