# Supplementary material for: A Rapid Screening Method of Candidate Probiotics for Inflammatory Bowel Diseases and the Anti-inflammatory Effect of the Selected Strain Bacillus smithii XY1
Source: Front Microbiol. 2021 Dec 17;12:760385. doi: 10.3389/fmicb.2021.760385 (PMC8718878; doi:10.3389/fmicb.2021.760385)
Supplement: Supplementary file 1 [file Data_Sheet_1.docx]

**Supplementary materials**

**Strain isolation method**

| **Strain** | **Species** | **Source** | **temperature** | **Culture medium** | **Oxygen environment** |
| --- | --- | --- | --- | --- | --- |
| GG | *Lactobacillus rhamnosus* | ATCC | 37 ℃ | MRS | Anaerobic |
| CGMCC1.4261 | *Paenibacillus polymyxa* | CGMCC | 37 ℃ | LB | Aerobic |
| DSM4216 | *Bacillus smithii* | CGMCC | 50 ℃ | LB | Anaerobic |
| NBRC12583 | *Bacillus coagulans* | CGMCC | 50 ℃ | MRS | Anaerobic |
| NBRC3425 | *Lactobacillus rhamnosus* | CGMCC | 37 ℃ | MRS | Anaerobic |
| CICC10061 | *Bacillus thuringiensis* | CICC | 37 ℃ | LB | Aerobic |
| CICC10309 | *Azotobacter chroococcum Beijerinck* | CICC | 37 ℃ | LB | Aerobic |
| CICC10580 | *Paenibacillus polymyxa* | CICC | 37 ℃ | LB | Aerobic |
| CICC20022 | *Lacobacillus plantarum subsp.plantarum* | CICC | 37 ℃ | MRS | Anaerobic |
| CICC20643 | *Bacillus subtilis subsp. subtilis* | CICC | 37 ℃ | LB | Aerobic |
| CICC23057 | *Bacillus cereus* | CICC | 37 ℃ | LB | Aerobic |
| CICC23632 | *Bacillus megaterium* | CICC | 37 ℃ | LB | Aerobic |
| XY1 | *Bacillus smithii* | Coffee grounds | 50 ℃ | MRS*/LB | Aerobic |
| XY2 | *Bacillus coagulans* | Coffee grounds | 50 ℃ | MRS | Aerobic |
| XY3 | *Bacillus smithii* | Coffee grounds | 50 ℃ | MRS*/LB | Aerobic |
| XY4 | *Bacillus smithii* | Coffee grounds | 50 ℃ | MRS*/LB | Aerobic |
| XY5 | *Bacillus smithii* | Coffee grounds | 50 ℃ | MRS*/LB | Aerobic |
| XY6 | *Bacillus smithii* | Coffee grounds | 50 ℃ | MRS*/LB | Aerobic |
| XY8 | *Bacillus coagulans* | Coffee grounds | 50 ℃ | MRS | Anaerobic |
| XY9 | *Aneurinibacillus danicus* | Coffee grounds | 50 ℃ | LB | Anaerobic |
| XY10 | *Aneurinibacillus danicus* | Coffee grounds | 50 ℃ | LB | Anaerobic |
| XY11 | *Aneurinibacillus danicus* | Coffee grounds | 50 ℃ | LB | Anaerobic |
| XY12 | *Bacillus subtilis* | Coffee grounds | 50 ℃ | LB | Aerobic |
| XY13 | *Bacillus subtilis* | Coffee grounds | 50 ℃ | LB | Aerobic |
| XY14 | *Bacillus subtilis* | Coffee grounds | 50 ℃ | LB | Aerobic |
| XY15 | *Bacillus subtilis* | Coffee grounds | 50 ℃ | LB | Aerobic |
| XY16 | *Bacillus licheniformis* | Coffee grounds | 50 ℃ | LB | Aerobic |
| XY17 | *Bacillus fumarioli* | Coffee grounds | 50 ℃ | LB | Aerobic |
| XY18 | *Bacillus fumarioli* | Coffee grounds | 50 ℃ | LB | Aerobic |
| XY19 | *Bacillus fumarioli* | Coffee grounds | 50 ℃ | LB | Aerobic |
| XY20 | *Bacillus ginsengihumi* | Coffee grounds | 50 ℃ | LB | Aerobic |
| XY21 | *Paenibacillus phoenicis* | Coffee grounds | 50 ℃ | LB | Aerobic |
| XY22 | *Bacillus thermoamylovorans* | Coffee grounds | 50 ℃ | LB | Aerobic |
| XYHYN | *Bacillus coagulans* | Dairy product | 50 ℃ | MRS | Anaerobic |
| XYRR2 | *lactobacillus parafarraginis* | Dairy product | 37 ℃ | MRS | Anaerobic |
| XYRR3 | *Lactobacillus paracasei* | Dairy product | 37 ℃ | MRS | Anaerobic |
| XYB | *Lactobacillus buchneri* | EM product | 37 ℃ | MRS | Anaerobic |
| XYR | *Lactococcus lactis subsp. Hordniae* | EM product | 37 ℃ | MRS | Anaerobic |
| XYT2 | *Bacillus subtilis* | EM product | 37 ℃ | LB | Aerobic |
| XYT3 | *Bacillus licheniformis* | EM product | 37 ℃ | LB | Aerobic |

**Culture medium: * means a strain can grow on it but it is not advised to be used as culture medium**

Table. S2 Source and accession number of 17 LAB and bacillus strains.

| Strains | Species | Source | Accession # |
| --- | --- | --- | --- |
| Strains passed first screening (growth ability test) | | | |
| CICC20022 | *Lactobacillus plantarum subsp.plantarum* | CICC | MW276127 |
| XYT2 | *Bacillus subtilis* | EM Product | MW276124 |
| XYT3 | *Bacillus licheniformis* | EM Product | MW276123 |
| XYRR3 | *Lacticaseibacillus paracasei* | Dairy product | MW276121 |
| XY16 | *Bacillus licheniformis* | Coffee grounds | MW276116 |
| XYR | *Lactococcus lactis subsp. hordniae* | EM product | MW276125 |
| XYB | *Lactobacillus buchneri* | EM product | MW276119 |
| XYHYN | *Bacillus coagulans* | Dairy product | MW276122 |
| XY12 | *Bacillus subtilis* | Coffee grounds | MW276115 |
| CICC20643 | *Bacillus subtilis subsp. subtilis* | CICC | MW276126 |
| Strains passed second screening (Anti-inflammatory test I) | | | |
| GG | *Lactobacillus rhamnosus* | ATCC | MG827277.2 |
| DSM4216 | *Bacillus smithii* | CGMCC | NR_118971.1 |
| XYRR2 | *Lactobacillus parafarraginis* | Dairy product | MW276120 |
| NBRC3425 | *Lactobacillus rhamnosus* | CICC | NR_043408 |
| NBRC12583 | *Bacillus coagulans* | CGMCC | NR_041523 |
| XY2 | *Bacillus coagulans* | Coffee grounds | MW276118 |
| Strains passed final screening (Anti-inflammatory test II) | | | |
| XY1 | *Bacillus smithii* | Coffee grounds | MW276117 |

**Assessment of acid and bile tolerance.**

**Method**

The method was carried out as described by a former paper with some modifications(Dang et al. 2018). Overnight cultures grown in MRS or LB broth at 37 °C were harvested by centrifugation (10 000 rpm, 5 min, 4 °C), then washed the precipitates twice with PBS and resuspended then in MRS or LB broth adjusted to pH 3.00 or broth supplemented with 0.3% bile salts. The mixture was then incubated at 37 °C. For acid tolerance assessment, the mixture was incubated for 2 h at 37°C. For bile tolerance assessment, the mixture was incubated for 4 h at at 37°C. The survival rate was calculated as the following equation.

Survival rate (%) = log CFU N_t_/log CFU N_0_Ⅹ100%

N_0_ was the total viable counts before treatment and N_t_ was the total viable counts after treatment.

**Resullt**


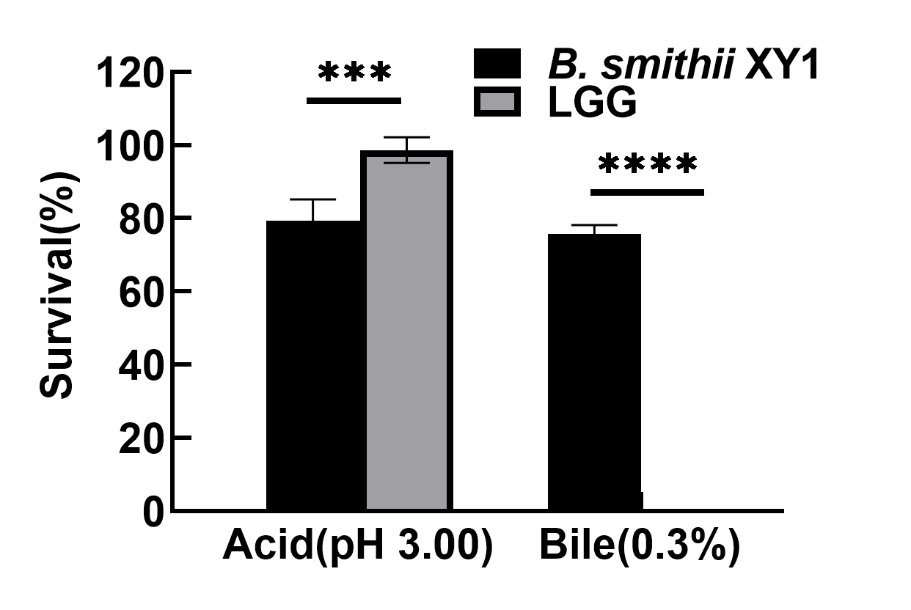


Figure. S1 Comparision of Acid and bile tolerance of *B.smithii* XY1 and LGG. Data are presented as mean ± s.e.m. ***p < 0.001, ****p < 0.0001, analyzed by two-tail test. 3 biological replicates were conducted.

Dang, Fangfang; Jiang, Yujun; Pan, Ruili; Zhou, Yanhong; Wu, Shuang; Wang, Rui et al. (2018): Administration of Lactobacillus paracasei ameliorates type 2 diabetes in mice. In Food & function 9 (7), pp. 3630–3639. DOI: 10.1039/c8fo00081f.
